# How the Lifestyle Leaders View and Manage Medications

**DOI:** 10.1093/geroni/igaa057.2072

**Published:** 2020-12-16

**Authors:** Taylor Patskanick, Julie Miller

**Affiliations:** 1 MIT, Cambridge, Massachusetts, United States; 2 MIT AgeLab, Cambridge, Massachusetts, United States

## Abstract

Medication management is an ongoing consideration for adults ages 85 and older, their caregivers, and healthcare providers. When asked about their attitudes and behaviors regarding medication management, over 73% of the Lifestyle Leaders reported taking 3+ prescription medications daily and managing their own medication regimes. 61.9% of participants had taken over-the-counter, non-prescription medication for pain over the past five years. When asked why some participants didn’t currently take prescription medications to manage pain, the most frequently-reported responses were: “I don’t feel that my pain warrants a prescription medication,” (19%, n=8), “I don’t want to deal with the side effects,” and “I don’t trust drug companies,” (9.5%, n=4, respectively). The Lifestyle Leaders reported they would be most likely to go to the internet (over their local pharmacist) to ask for advice about their medication(s). Meanwhile, 39% of Lifestyle Leaders would trust a robot to manage their medication(s) for them.

